# Assessing the Societal Costs of Cognitive Aging: Evidence From the US and Mexico

**DOI:** 10.1093/geroni/igaf122.598

**Published:** 2025-12-31

**Authors:** Flavia Andrade, Jacqueline Angel

## Abstract

As the United States and Mexico undergo demographic shifts, rising longevity has intensified the burden of chronic conditions such as dementia and diabetes. These conditions pose significant policy and financial challenges, particularly as families bear most caregiving costs. This symposium advances knowledge on caregiving in the U.S. and Mexico, using diverse data sources to examine barriers and facilitators in chronic disease management. Five papers reflect collaborations between emerging scholars and senior faculty from the International Conference on Aging in the Americas. Falk analyzes BRFSS data, showing that caregivers of individuals with Alzheimer’s Disease and related dementias (ADRD), especially women and those facing food insecurity, experience significantly higher stress, underscoring the need for targeted interventions. Rojas-Alvarez uses HEPESE and Medicare data to show that Hispanic older adults with dual diagnoses of diabetes and ADRD near the U.S.-Mexico border experience worse healthcare utilization and self-reported health, pointing to structural barriers. Bokum analyzes the HEPESE and ACS to examine financial strain among co-residing adult child caregivers, finding younger and late-middle-aged caregivers face disproportionate financial stress, emphasizing the need for direct compensation and expanded support. Aguila draws on MHAS and HRS sister surveys to assess informal dementia care, identifying public and family-based care gaps requiring policy action. Mudrazija and Aranda estimate that unpaid dementia caregiving costs will rise substantially by 2060, disproportionately affecting minoritized caregivers and highlighting the need for culturally tailored support. Together, these studies inform strategies to enhance caregiver support and reduce health and financial disparities.

